# Antitumor Activity of a Mesenchymal Stem Cell Line Stably Secreting a Tumor-Targeted TNF-Related Apoptosis-Inducing Ligand Fusion Protein

**DOI:** 10.3389/fimmu.2017.00536

**Published:** 2017-05-11

**Authors:** Irene Marini, Martin Siegemund, Meike Hutt, Roland E. Kontermann, Klaus Pfizenmaier

**Affiliations:** ^1^Institute of Cell Biology and Immunology, University of Stuttgart, Stuttgart, Germany

**Keywords:** mesenchymal stem cells, apoptosis, non-viral transfection, TNF-related apoptosis-inducing ligand, diabody, cell-based therapy, mouse xenograft

## Abstract

Mesenchymal stem cells (MSCs) are currently exploited as gene delivery systems for transient *in situ* expression of cancer therapeutics. As an alternative to the prevailing viral expression, we here describe a murine MSC line stably expressing a therapeutic protein for up to 42 passages, yet fully maintaining MSC features. Because of superior antitumoral activity of hexavalent TNF-related apoptosis-inducing ligand (TRAIL) formats and the advantage of a tumor-targeted action, we choose expression of a dimeric EGFR-specific diabody single-chain TRAIL (Db-scTRAIL) as a model. The bioactivity of Db-scTRAIL produced from an isolated clone (MSC.TRAIL) was revealed from cell death induction in Colo205 cells treated with either culture supernatants from or cocultured with MSC.TRAIL. *In vivo*, therapeutic activity of MSC.TRAIL was shown upon peritumoral injection in a Colo205 xenograft tumor model. Best antitumor activity *in vitro* and *in vivo* was observed upon combined treatment of MSC.TRAIL with bortezomib. Importantly, *in vivo* combination treatment did not cause apparent hepatotoxicity, weight loss, or behavioral changes. The development of well characterized stocks of stable drug-producing human MSC lines has the potential to establish standardized protocols of cell-based therapy broadly applicable in cancer treatment.

## Introduction

Mesenchymal stem cells (MSCs) are multipotent stem cells that have generated a great deal of interest since their first identification in 1960s by Friedenstein (1, 2) due to their exceptional capabilities, foremost multilineage differentiation potential (3, 4), and hypoimmunogenic properties (5, 6). Because of these features, MSCs were early on applied in the field of regenerative medicine (7) and subsequently in a variety of other diseases, including autoimmune disorders, cardiovascular malignancies, and liver diseases [reviewed by Squillaro et al. (8)].

In addition, because of their tumor-homing capability [reviewed by Hagenhoff et al. (9)], MSCs are currently exploited as cell-based delivery systems for cancer protein therapeutics (10, 11). Conceptionally, it is anticipated that through tumor homing of MSC the localized production of a given therapeutic protein is advantageous over systemic application of a recombinant protein considering not only effective *in situ* concentrations of the drug and thus favorable pharmacokinetic parameters but also minimizing unwanted systemic actions, often being the dose-limiting factor in clinical application. The TNF-related apoptosis-inducing ligand (TRAIL), also known as Apo2L (12), has raised great hopes for a novel, broadly applicable treatment of cancers due to its apparently selective induction of tumor cell apoptosis. However, the clinical trials with a recombinant soluble form of TRAIL, consisting of a non-covalently assembled homotrimer, by and large, failed to show therapeutic activity (13, 14), whereas inadvertently existing agglomerates in preparations of soluble TRAIL displayed toxic activity toward non-malignant tissue, in particular hepatocytes (15). Over the past decades, many recombinant versions of TRAIL have been generated to enhance its pharmacokinetics and/or antitumoral activity (16–18). By now, it is evident that the failure of a soluble, strictly trimeric TRAIL in clinical trials is not only due to very short serum half-life but even more related to the fact that proper death receptor activation requires stable receptor crosslinking, which can be achieved by at least a hexavalent organization of the TRAIL molecule (19). Nevertheless, to cope with insufficient pharmacokinetic properties, several studies have addressed the use of *in situ* production of a standard soluble TRAIL molecule by different adult stem cells (20–22). Further, two studies have reported antitumoral activity of human MSC expressing antibodies in a diabody format (23, 24).

So far, use of viral vectors prevails to introduce therapeutic genes into stem cells, despite still existing safety concerns [reviewed by Stuckey and Shah (25)] because, conceptionally, viral transduction allows the use of autologous, patient-derived stem cells for gene delivery. However, due to the apparently low immunogenicity of MSCs, allogeneic transplantation is effectively used in regenerative medicine (26, 27) and, thus allows an alternative concept for *in situ* cell-based production of protein therapeutics. Based on these considerations and on knowledge about the requirements of effective apoptosis induction by TRAIL ligands, we investigated whether it is possible to generate a MSC line stably producing a highly bioactive, tumor-targeted single-chain TRAIL fusion protein under retention of its full MSC properties. Here, we report on the establishment of such a cell line (MSC.TRAIL) and its therapeutic activity in a xenotransplantation tumor model.

## Materials and Methods

### Cell Lines

Mouse bone marrow-derived MSC have been previously described (28) and were kindly provided by Dr. Angelika Hausser (IZI, University of Stuttgart, Germany). These cells were cultivated under sterile conditions, at 37°C in a 5% CO_2_ humidified atmosphere, in alpha-MEM supplemented with 10% FBS (HyClone) plus 1% penicillin/streptomycin. MSCs were passaged at a confluence of 70% every 3–4 days if not mentioned otherwise. Colo205 and HCT116 cells were obtained from ATCC (Manassas, VA, USA) and cultured, at 37°C and 5% CO_2_, in RPMI-1640 medium (Invitrogen) supplemented with 10% FCS (Thermo Fisher Scientific).

### MSC Transfection

Mesenchymal stem cells were transfected with polyethylenimine (PEI) using a ratio 1:3 for DNA and PEI. Briefly, 150 × 10^3^ cells/well in a six-well plate were grown in 2-ml culture medium for 18 h. Next, cell culture medium was removed, and 1.5 ml of serum-free alpha-MEM was added. Three hundred microliters of Opti-MEM were incubated with 12 μg of PEI for 5 minutes (min) at room temperature (RT). Next, 4 μg of plasmid DNA was added to the mixture, and after 20 min incubation, the mix was carefully added drop-wise to the cells. After 18 h incubation at 37°C cells were transferred into a flask and allowed to grow in cell culture medium for 24 h. Next, in order to select the transfected cells, 250 μg/ml of geneticin (G418) was added to the medium. Subsequently, a single clone selection, making limiting dilutions with a statistical density of 1 cell/well was performed. The best clone, named MSC.TRAIL, was used for further studies. The coding sequence of Db-scTRAIL (EGFR-specific pCR3-Db-scTRAIL) construct was reported by Siegemund et al. (19).

### Purification of Recombinant Proteins

The EGFR targeting Db-scTRAIL fusion protein (see Figure S1 in Supplementary Material) was produced from transfected MSCs and purified from cell culture supernatant by anti-FLAG affinity chromatography as described previously (19). In brief, cell-free supernatant was incubated with anti-FLAG M2 Affinity Gel (0.3 ml bead volume/100 ml supernatant, Sigma-Aldrich) for at least 2 h or alternatively overnight at 4°C on a roller mixer, prior to collecting of beads in an empty column, washing with TBS, and eluting with 100 μg/ml FLAG peptide in TBS. After dialysis in PBS, eluates were concentrated with Vivaspin 20 devices (50 kDa, Sartorius), and the purified protein was analyzed by western blotting.

### Cell Death Assays

Colo205 (4 × 10^4^ cells/well), HCT116 (3 × 10^4^ cells/well), or MSCs (2 × 10^4^ cells/well) were grown in 100-μl culture medium in 96-well plates for 18 h, followed by treatment either with serial dilutions of Db-scTRAIL proteins or supernatant from transfected MSCs, in triplicates. For positive control, cells were killed with 0.25% Triton X-100. Cell death assays were performed in the absence or presence of bortezomib (BZB) (250 ng/ml; UBPBio). TRAIL blocking antibody (1 μg/ml; Enzo Life Sciences) was used in the combined treatments. BZB was added 30 min before incubation with the proapoptotic proteins to sensitize cancer cells for cell death induction. After 16 h of incubation, cell viability was determined by crystal violet staining (19).

### Coculture of MSCs and Cancer Cells

Colo205 (1 × 10^4^ cells/well) or HCT116 (8 × 10^3^ cells/well) were seeded in 24-well plates, in 600 μl of MSC medium (alpha-MEM), and allowed to grow at 37°C. After overnight incubation, MSCs were added using different ratios of MSCs:colorectal cancer (CRC) cells, in a final volume of 1 ml/well. Different treatments with BZB (250 ng/ml) and TRAIL blocking antibody (1 μg/ml) were performed and finally analyzed by crystal violet staining as described above.

### Enzyme-Linked Immunosorbent Assay (ELISA)

Enzyme-linked immunosorbent assay assays were performed using the kit OptEIA™ human TRAIL ELISA Set (BD), according to the manufacturer’s instruction. Briefly, the ELISA plate was coated with 100 μl/well of capture antibody and incubated overnight at 4°C, and the remaining binding sites were blocked with 2% (w/v) dry milk/PBS. Next, a titration (1:3) of standard TRAIL protein and either serial dilutions of MSC supernatant or serum blood (dilution 1:20) were added and incubated for 2 h at RT, in duplicate. After five washing steps, working detector solution was incubated for 1 h at RT. Bound proteins were detected using ELISA developing solution (0.1 mg/ml TMB, 100 mmol/l sodium acetate buffer, pH 6.0, 0.006% H_2_O_2_). The reaction was stopped with H_2_SO_4_ (1 mol/l). Absorbance was measured at 450 nm in an ELISA reader.

### Western Blotting

TNF-related apoptosis-inducing ligand secreted by transfected MSCs in culture medium was purified by an anti-FLAG affinity chromatography as described above. The purified proteins were separated on SDS-PAGE (12%) and then blotted on PVDF membrane. After incubation with primary antibody (anti-TRAIL MAB687, R&D), the secondary anti-mouse HRP-conjugated antibody (Sigma-Aldrich) was added. Finally, the membrane was treated with a peroxidase substrate (enhanced chemiluminescence detection system from Pierce) according to the manufacturer’s instructions to visualize the signals and exposed to an X-ray film.

### Flow Cytometry

To analyze the expression of surface markers, MSCs (10 × 10^4^ cells/well) were seeded in a 96-well plate and incubated with the directly conjugated antibody (CD9, CD44, CD71, CD105, CD14, and CD34). Signals from respective isotype control antibodies were subtracted from all samples to compensate unspecific antibody binding.

Propidium iodide (PI, Sigma-Aldrich) staining of cells was done after 18 h of treatment. The cells were collected, incubated with 10 μg/ml of PI, and immediately analyzed by flow cytometry.

In order to test cleaved caspase-3 activation, Colo205 (8 × 10^4^ cells/well) were seeded in the bottom of a transwell plate (Costar) and incubated at 37°C overnight. Then, BZB (250 ng/ml) was added into the culture medium and incubated for 30 min. Next, into the upper chamber of the transwell, 16 × 10^3^ cells for each MSC line were seeded. After 18 h of coculture, Colo205 cells from the lower chamber were collected, fixed in PFA (4%), and permeabilized with 0.1% Triton X-100. Then, primary antibody (Asp 175, Cell Signaling Technology) was incubated for 1 h at RT. After two washing steps and secondary antibody incubation, the cells were resuspended and analyzed.

### Immunofluorescence and Microscopy

Mesenchymal stem cells were seeded on glass coverslips and incubated for 3 h at 37°C. Then, coverslips were washed with PBS and cells were fixed with 4% PFA and permeabilized with 0.1% Triton X-100. The blocking step was performed by incubating the cells with 5% FBS in PBS for 30 min at RT. Next, cells were washed and incubated with primary antibody and DAPI (1 μg/ml, Sigma) diluted in blocking buffer for 2 h. When required, incubation with secondary antibody, diluted in blocking buffer, was performed for 1 h. Coverslips were mounted in Fluoromount G and analyzed with the Spinning Disc (Zeiss) using 488, 543, and 633 nm excitation and a 20×/0.8 DIC objective lens. Images were processed with ZEN software.

### Adipogenic and Osteogenic Differentiation

For the adipogenic differentiation, MSCs were grown to confluence on Permanox 4-well chamber slides (Thermo Scientific). Then, the culture medium was replaced with adipogenic medium (α-MEM supplemented with 10% FBS, 1% penicillin/streptomycin, 1 μM dexamethasone, 500 μM IBMX, 10 μg/ml human insulin, and 100 μM indomethacin) and changed every 2–3 days. Twelve days after initial adipogenic induction, cells were washed with PBS and fixed for 10 min in 4% Histofix (Roth). Next, cells were rinsed once with H_2_O, incubated in 60% isopropanol for 5 min, and then with Oil Red O for 10 min. Finally, the cells were washed once with 60% isopropanol followed by H_2_O. Nuclei were counterstained with hemalaun. As a negative control, cells grown in culture medium for 12 days were used. In order to analyze the osteogenic differentiation, MSCs were grown to 90–100% confluence in 24-well plates and then cultured in osteogenic medium (α-MEM supplemented with 15% FBS, 1% penicillin/streptomycin, 100 nM dexamethasone, 50 μg/ml ascorbate-2- phosphate, and 10 mM beta-glycerol phosphate) for 21 days. At this time point, the differentiation was assessed by Alizarin Red staining. In brief, cells were washed with PBS and allowed to dry for 5–10 min. Next, cells were fixed with 50% ethanol for 20 min. The fixed cells were then stained with 1% Alizarin red (Roth) at pH 6.4 for 30 min under continuous shaking. Finally, cells were rinsed three times with H_2_O, and transmitted light pictures were taken. As a negative control, cells grown in culture medium for 21 days were used.

### Xenograft Mouse Tumor Model

Animal care and all experiments performed were in accordance with federal guidelines and had been approved by university and state authorities. Female NMRI nu/nu mice (Janvier), 8 weeks old, were injected subcutaneously (s.c.) with 3 × 10^6^ Colo205 cells in 100 μl PBS at left and right dorsal sides. Treatment started 10 days after tumor cell inoculation when tumors reached ~100 mm^3^. In particular, 4 × 10^6^ MSCs were resuspended in 100 μl PBS mixed with 100 IU/ml of heparin (29) and then peritumorally injected (p.t.). During the injections of all cell lines, mice were anesthetized with isoflurane. The Colo205-bearing mice received three p.t. injections of MSCs at day 10, 17, and 27. In addition, 5 μg of BZB in 100 μl PBS were injected i.p. every second day, starting from day 11 until day 31. Mice in the control groups received either 100 μl PBS i.p. injected or MSCs.Mock. Tumor growth was monitored as described (30). Blood samples were taken from the tail at day 31, centrifuged (10,000 × *g*, 10 min, 4°C) and then stored at −80°C. Activity of ALT was determined by an enzymatic assay accordingly to the manufacturer’s instructions (BIOO Scientific, Austin, TX, USA).

### Statistics

All values are expressed as means ± SD, while for the analysis of the *in vivo* studies the 95% confidence interval (95% CI) was used. Significances, for each experiment, were calculated with GraphPad prism one-way ANOVA with Tukey’s post-test. In particular: * represents a *p*-value < 0.05, ** represents a *p*-value < 0.01, and *** represents a *p*-value < 0.001.

## Results

### Engineered MSCs Express Bioactive Soluble Db-scTRAIL

**Figure 1:**
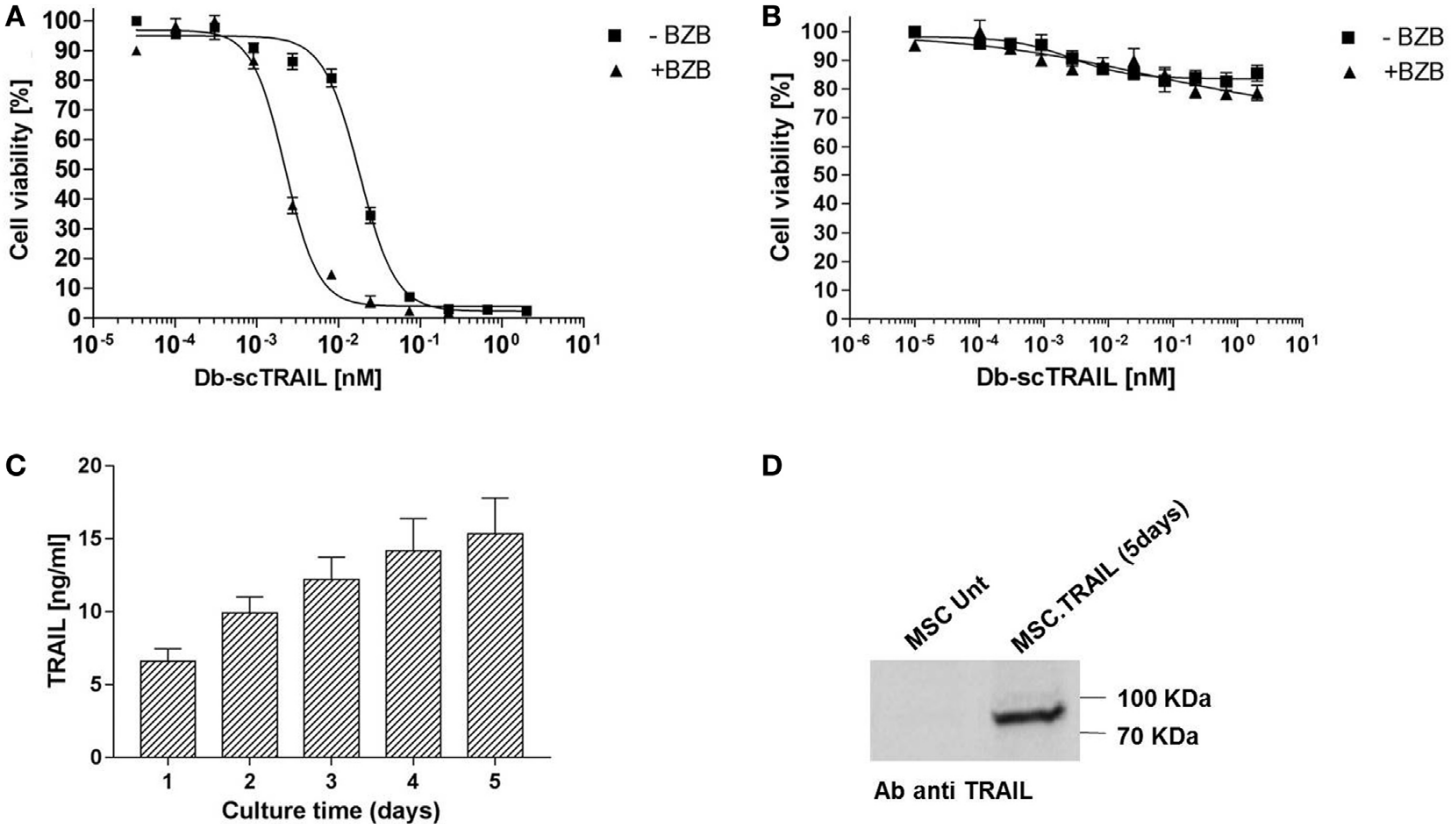
**Sensitivity of Colo205 and mesenchymal stem cells (MSCs) to diabody single-chain TNF-related apoptosis-inducing ligand (Db-scTRAIL) activity**. **(A)** Colo205 cells and **(B)** MSCs were treated with serial dilutions (titration 1:3) of Db-scTRAIL in the absence (filled squares) or in the presence (filled triangles) of 250 ng/ml of bortezomib (BZB). After 18 h, cell viability was determined using crystal violet staining. Data were normalized using BZB-treated cells or cells treated with normal medium for Db-scTRAIL + BZB or Db-scTRAIL alone, respectively (mean ± SD, *n* = 3). **(C)** MSCs were transiently transfected (PEI), and the amount of soluble Db-scTRAIL released in cell culture medium was measured by enzyme-Linked Immunosorbent Assay, every 24 h (mean ± SD, *n* = 3). **(D)** After 5 days of transient transfection, Db-scTRAIL secreted in cell medium was purified and analyzed by western blotting using a specific antibody against TRAIL (MSC.Unt: MSC untransfected, MSC.TRAIL: MSC transfected with TRAIL).

**Figure 2:**
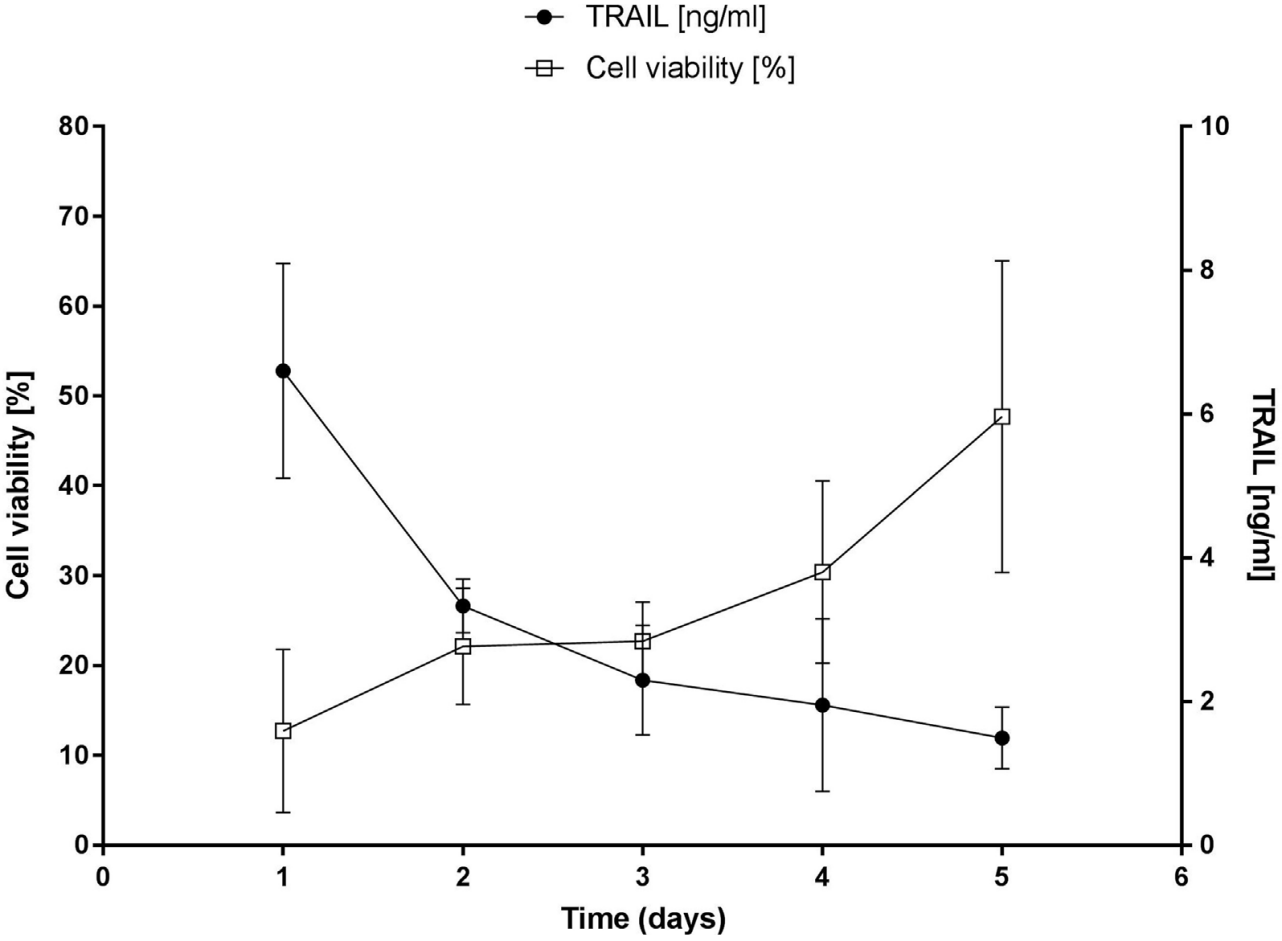
**Cell death induction assay of secreted diabody single-chain TNF-related apoptosis-inducing ligand (Db-scTRAIL) on Colo205**. Left axis: Colo205 cells were sensitized with bortezomib (BZB, 250 ng/ml) and treated with an aliquot of daily collected and renewed medium (dilution 1:3) from transiently transfected mesenchymal stem cells (MSCs). After 18 h of treatment, cell viability of Colo205 was determined using crystal violet staining and data were normalized using BZB-treated cells as control (mean ± SD, *n* = 4). Right axis: the daily amount of soluble Db-scTRAIL released in cell culture medium of MSCs was measured by enzyme-linked immunosorbent assay (mean ± SD, *n* = 3).

As a prerequisite to study the application of MSCs as cell-based therapy for Db-scTRAIL fusion protein expression, we first investigated the TRAIL sensitivity of these cells in comparison to a CRC cell line Colo205. In accordance with our previous study (19, 30), combinatorial activity of the Db-scTRAIL fusion protein with the apoptosis sensitizer BZB exerts a potential superior apoptotic effect on CRC cells. In fact, we observed a strong enhancement of cell death induction on Colo205 cells upon combined treatment resulting in a ~9-fold increase of TRAIL-mediated apoptosis induction with BZB (EC_50_ values: Db-scTRAIL 19 pM; Db-scTRAIL + BZB 2.2 pM) (Figure 1A). Conversely, under the same conditions, MSCs were fully resistant to Db-scTRAIL activity even in the presence of the sensitizer (Figure 1B), confirming that MSCs are a well-suited cell delivery system of highly active TRAIL variants. Next, we tested Db-scTRAIL expression after transient transfection by ELISA and immunoblotting as well as *in vitro* bioactivity. MSCs were able to secrete Db-scTRAIL, revealing an accumulation of intact product over the observed time period of 5 days (Figures 1C,D). With the applied transient transfection protocol, Db-scTRAIL production, as revealed by specific ELISA and bioassay (induction of Colo205 cell death) was highest 1 day after transfection (~0.44 pg/cell) and declined thereafter, with still significant protein and bioactivity detectable after 5 days of culture and four subsequent media changes (Figure 2). Additionally, we tested cell death induction with PI staining after coculture of MSCs and Colo205 at ratios of 1:5 and 1:50, showing a significant increase of PI levels in a cell-dose and BZB-dependent manner (Figure S2 in Supplementary Material). These data collectively demonstrate that MSCs are a suitable system for the expression of bioactive Db-scTRAIL proteins.

### Stable MSCs.TRAIL Cell Line Induce Tumor Apoptosis by Caspase 3 Activation *In Vitro*

**Figure 3:**
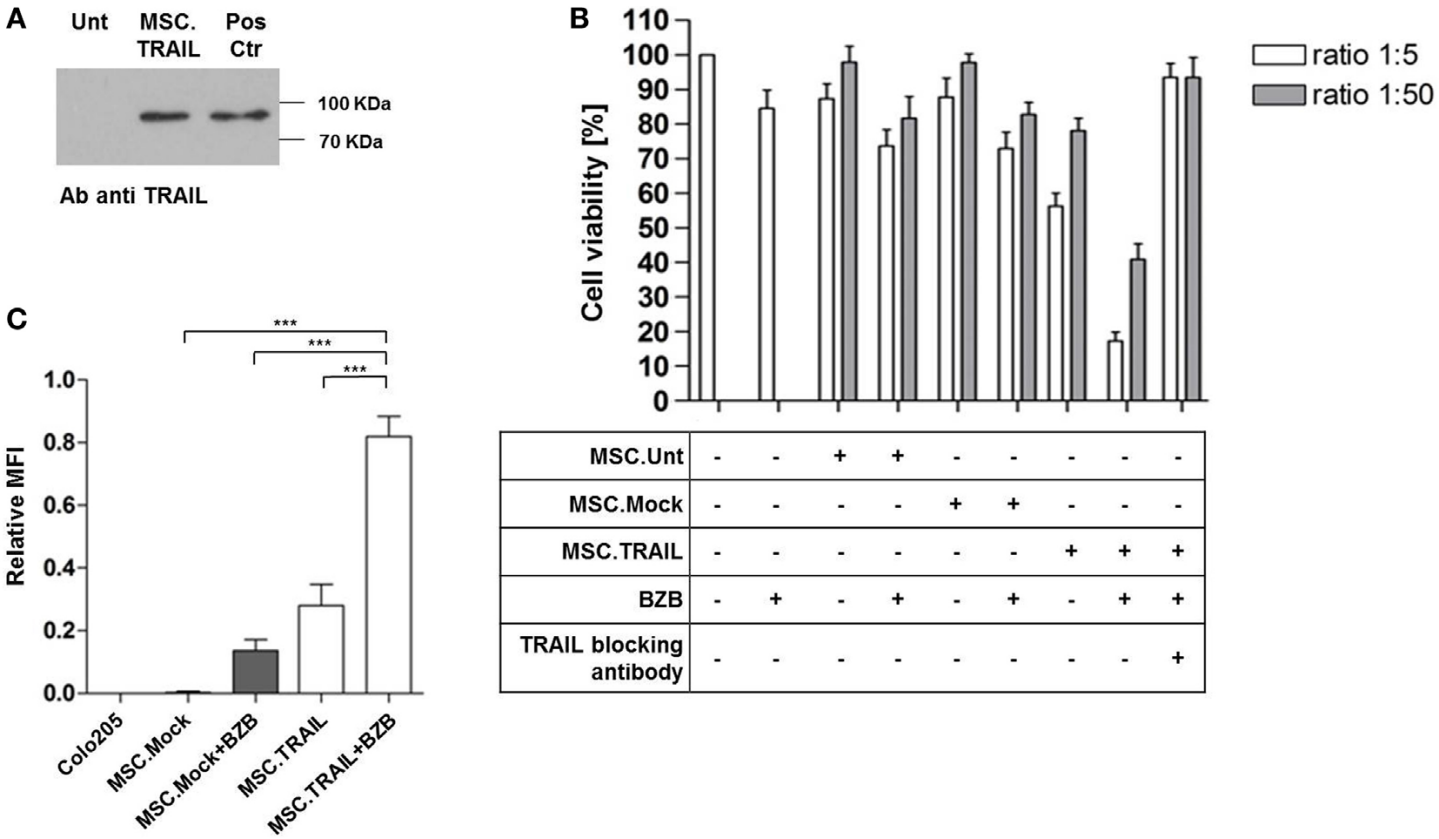
**Diabody single-chain TNF-related apoptosis-inducing ligand (Db-scTRAIL) released by mesenchymal stem cell (MSC).TRAIL cell line induces specific apoptotic activity in Colo205**. **(A)** Immunoblotting of purified Db-scTRAIL from MSC untransfected (Unt) and MSC.TRAIL cell culture medium (5 days), using a specific antibody anti TRAIL. Three micrograms of purified Db-scTRAIL were used as positive control (Pos Ctr). **(B)** The bioactivity of the secreted Db-scTRAIL was tested after 18 h of coculture of MSC lines and Colo205 (ratios 1:5 and 1:50). The cocultures were treated in combination with bortezomib (BZB) (250 ng/ml) and/or TRAIL blocking antibody (1 μg/ml). Cell viability was analyzed using crystal violet staining and data were normalized using Colo205 cells treated with BZB as control (mean ± SD, *n* = 3). **(C)** Colo205 cells were seeded in the lower chamber of a transwell (8 × 10^4^ cells). After overnight cultivation, the stable cell lines (Mock.TRAIL and MSC.TRAIL) were seeded in the upper chamber (1.6 × 10^4^ cells) and BZB (250 ng/ml) was added to the medium. After 18 h of treatment, Colo205 were collected, stained with the specific cleaved caspase-3 antibody (Asp 175), and analyzed by flow cytometry (mean ± SD, *n* = 4).

Based on the positive results with transient transfection of MSCs, we aimed at generation of stable producer clones by standard selection methods and isolation of individual clones by limiting dilution. The two highest expressing clones out of 13 identified positive clones after the first screening round proofed to be stable expressors *in vitro* and one clone, named MSC.TRAIL was used for further analyses of long-term expression and *in vivo* activity (Figure S3 in Supplementary Material). MSC.TRAIL showed cumulative secretion of the protein during culture for 5 days and stable expression of the product *in vitro* for 44 passages (data not shown). Western blot analysis of the purified TRAIL verified secretion of full-length protein (Figure 3A). Bioactivity of the secreted Db-scTRAIL was tested in coculture assays with Colo205 as target cells in the presence or absence of BZB (Figure 3B). A strong reduction of the cell viability in combination with BZB and complete neutralization of cell death in the coculture by a TRAIL blocking antibody proofed secretion of bioactive protein and strictly TRAIL-dependent cell death. The same results were observed after coculture of MSC.TRAIL with a different CRC cell line (HCT116) sensitive to TRAIL (Figures S4A,B in Supplementary Material). In order to confirm that the observed reduction of cell number is due to an apoptotic process, we analyzed cleaved caspase-3 levels as a hallmark of apoptosis induction. For this, we performed coculture using a double chamber system with a membrane allowing free exchange of soluble mediators. Colo205 cells were seeded in the bottom chamber and the MSC.TRAIL or the Mock cells were seeded in the upper chamber. Cleaved caspase-3 levels in Colo205 cells were determined by flow cytometry after 18 h of coculture (Figure 3C). As expected, a strong increase of cleaved caspase-3 levels was found when Colo205 cells were exposed to Db-scTRAIL-producing cells in combination with the sensitizer BZB. MSC.Mock served as negative control and a weak signal was noted upon incubation with sensitizer BZB only. Collectively, the *in vitro* studies show that stable MSC producer clone can be established exerting long-term expression of a highly bioactive Db-scTRAIL fusion protein.

### MSC Properties Are Not Affected by Stable Transfection *In Vitro*

**Figure 4:**
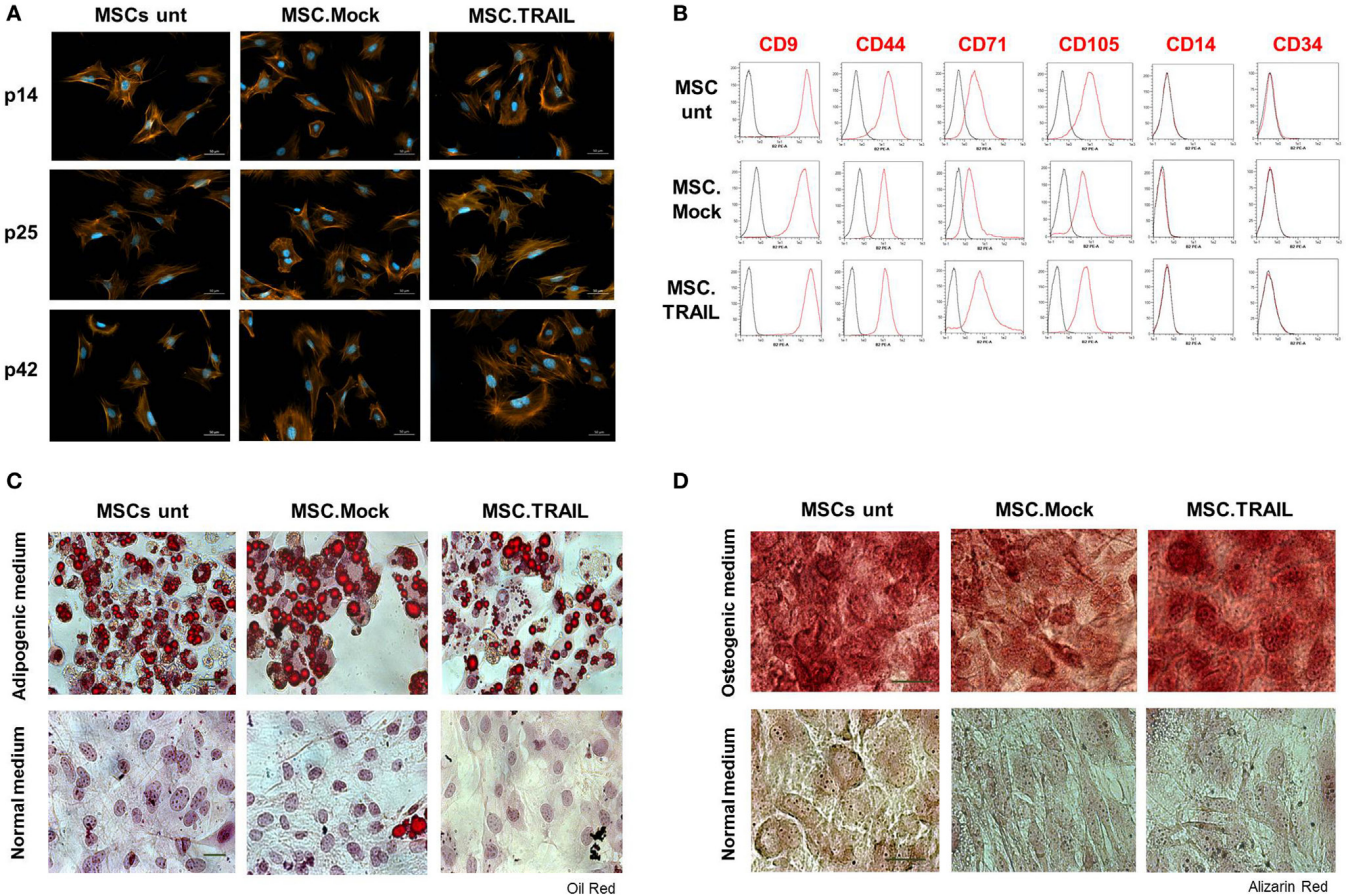
**Characterization of stable mesenchymal stem cell (MSC) lines**. **(A)** Mesenchymal phenotype of MSCs: untransfected (MSCs unt), MSC.TNF-related apoptosis-inducing ligand (TRAIL), and Mock (MSC.Mock) was analyzed at early (p. 14), middle (p. 25), and late (p. 42) passages. Cells were stained with Alexa Fluor568-coupled phalloidin (red/orange). The nuclei were counterstained with DAPI (blue). Scale bar, 50 μm. **(B)** Analysis of surface markers expression on MSC lines at passage 25. Cells were stained with indicated antibodies and binding was analyzed by flow cytometry (red line). Unstained cells were used as negative control (black line). *y*-Axis: number of events analyzed. Representative experiment out of five independent experiments performed. All cell lines (MSCs unt, MSC.Mock, and MSC.TRAIL) were cultured *in vitro* in **(C)** adipogenic or **(D)** osteogenic media, at passage 22. The cells were fixed and stained with Oil Red O (adipogenesis) or Alizarin Red (osteogenesis) for adipocyte or osteoblast differentiation. Cells cultured in normal medium were use as control. Scale bars = 50 μm (adipogenesis) and 100 μm (osteogenesis).

In order to investigate whether the transfection and isolation of a stable producer cell line affected MSC characteristics *in vitro*, we analyzed the properties of this cell line at different passages and compared it to mock-transfected and untransfected MSCs. We first tested the phenotype of MSCs by staining the cells with phalloidin to visualize the F-actin. All cell lines (MSCs untransfected, MSC.Mock, and MSC.TRAIL) displayed a typical spindle-shaped phenotype as described for MSCs (31). Remarkably, the phenotype did not change during *in vitro* cultivation up to passage 42 (Figure 4A). Next, we investigated the expression of stem cell markers. In accordance with the International Society of Cellular Therapy, all MSC lines analyzed were positive for CD9, CD44, CD71, and CD105 and lacked the expression of CD14 and CD34, as show in Figure 4B. No differences between untransfected MSCs and the stably transfected cell lines were observed. Interestingly, the pattern of marker expression was maintained from early passage (p9) up to passage 42 (Figure S5 in Supplementary Material) and is in accordance with murine MSCs lines described by others [reviewed by Boxall and Jones (32)]. Finally, we verified the multilineage differentiation capability of MSCs, which is the most characteristic feature. All MSC lines showed, on one hand, the capability to generate lipid droplets which indicate a successful adipogenic differentiation (Figure 4C). In rare cases, a spontaneous adipogenic differentiation was observed, probably due to a high cell density in the differentiation cultures, without a statistically significant frequency. Further, the cell lines were also able to display mineralization, observed by Alizarin red staining, confirming osteogenic differentiation ability (Figure 4D). The same results were observed at late passages for all cell lines (data not shown). These findings demonstrate that the stable non-viral transfection and the constitutive Db-scTRAIL secretion do not alter the typical properties of MSCs, even during long-term *in vitro* culture.

### MSC.TRAIL Exert a Significant Antitumor Activity in Combination with BZB *In Vivo*

**Figure 5:**
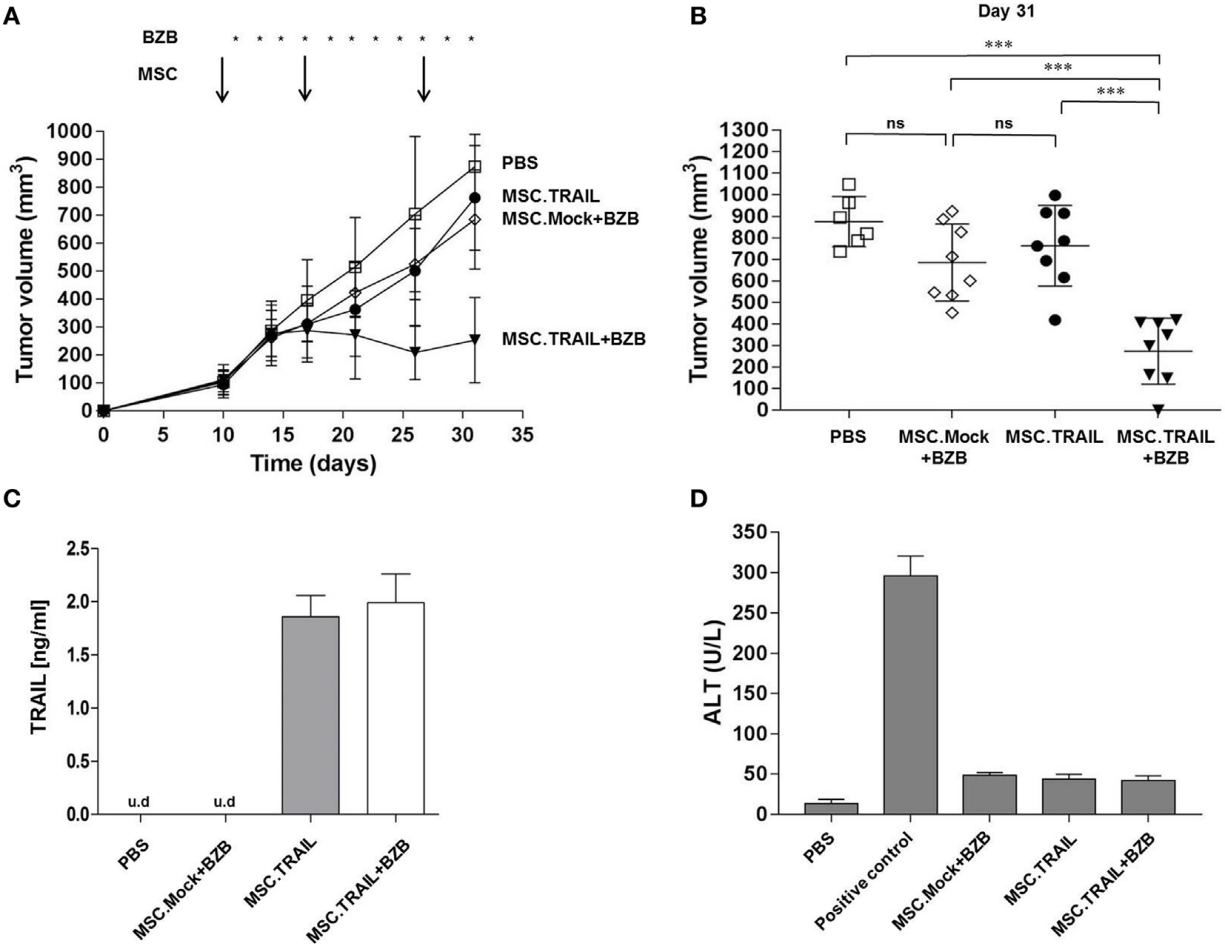
**Antitumor activity of mesenchymal stem cell (MSC).TNF-related apoptosis-inducing ligand (TRAIL) in a Colo205 xenograft tumor model**. **(A)** Tumor volume was analyzed as a function of time after p.t. injection of: PBS (squares), MSC.Mock + BZB (diamonds), MSC.TRAIL (filled circles), or MSC.TRAIL + BZB (filled triangles). Arrows, MSC p.t. administrations (4 × 10^6^ cells/injection on day 10, 17, and 27); asterisks, BZB application (5 μg every second day); *n* = 8 tumors/treatment group. **(B)** Individual tumor volumes at day 31 (*n* = 6 tumors for PBS group and *n* = 8 tumors for MSC.Mock + BZB, MSC.TRAIL, and MSC.TRAIL + BZB). Bars, mean of tumor volumes ± 95% confidence interval (CI) (n.s: not significant; ***p < 0,001). **(C)** Diabody single-chain TRAIL mouse serum levels were analyzed at day 26 by enzyme-linked immunosorbent assay assay (mean ± SD, *n* = 3; u.d, under detectable level). **(D)** Alanine aminotransaminase (ALT) activity was analyzed in mouse serum, at day 31, after three MSC p.t. injections (mean ± SD, *n* = 3). Positive control, 0.1 nmol Fas ligand fusion protein; negative control, PBS.

Based on our *in vitro* results, we assessed the potential therapeutic utility of MSC.TRAIL *in vivo*. First, we performed single subcutaneous injection of MSCs (s.c.; 4 × 10^6^ cells) in nude mice in order to verify the presence of Db-scTRAIL in the serum fraction after 1, 3, 7, 14, and 21 days by ELISA (Figure S6 in Supplementary Material). No TRAIL signals could be detected up to 3 days after injection, whereas after 7 and 14 days specific TRAIL signals were identified in the range of 1.5 ng/ml for MSC.TRAIL cells. As expected, all control cell injections gave no positive signal. Next, we investigated the antitumor activity of MSC.TRAIL in a Colo205 mouse xenograft tumor model, in which MSCs were peritumorally (p.t.) injected at three time points. The treatments started when tumors were palpable and vascularized, reaching a volume of ~100 mm^3^. At this time point, the first MSC (4 × 10^6^ cells) injection was performed, with Mock cells and PBS used as controls. In the combination treatment groups (MSC.TRAIL + BZB and MSC.Mock + BZB), 5 μg of BZB was intraperitoneally (i.p.) injected every other day. Up to 10 days after the first MSC injection, we observed no differences in tumor growth for all groups. However, from day 17 on, coincident with the second MSC administration, a slight, but increasingly significant reduction of tumor size was observed for the combination treatment group MSC.TRAIL + BZB over the whole observation period (31 days) (Figure 5A). Importantly, at day 26, serum analysis of Db-scTRAIL in animals receiving MSC.TRAIL revealed a concentration of ~1.5 ng/ml (Figure 5C) supporting a direct correlation of TRAIL activity and tumor reduction. Tumor response was maintained with the third application of MSC.TRAIL, although complete remission was achieved only in one case (Figure 5C). The observed MSC.TRAIL-dependent tumor response required co-administration of a low dose of the sensitizer BZB, corroborating the *in vitro* data. Thus, under the applied protocol, the MSC.TRAIL cells alone showed only a small, non-significant reduction in tumor growth, similar to the Mock-transfected MSC in combination with BZB (Figure 5B).

### Administration of MSC.TRAIL *In Vivo* Does Not Induce Acute Side Effects

In order to get insights into potential off-target, systemic side effects of continuous Db-scTRAIL expression in tumor-bearing animals, we analyzed serum levels of the liver enzyme alanine aminotransaminase (ALT) as an established marker of acute liver toxicity. ALT serum levels were determined in tumor-bearing mice at day 31, after three MSC p.t. injections. The analysis showed for all MSC groups only slightly elevated serum ALT levels compared to PBS group (<50 U/l), but low compared to the Fas ligand treatment known to cause acute liver toxicity (Figure 5D). This result revealed that the applied treatment protocol (three MSC administrations with and without BZB) did not induce discernable acute hepatotoxic effects *in vivo*, in accordance with a recent study from Yan and colleagues (21). Additionally, all the other standard parameters, such as body weight, remained in the normal range for the entire period of treatment (data not shown).

## Discussion

In this study, we explored the possibility of generating stable MSC producer cell lines as delivery system for the expression of an antitumor protein drug, using a tumor-targeted variant of the proapoptotic protein TRAIL as a model substance. Since its first identification (33, 34), TRAIL was extensively studied due to its characteristics of inducing apoptosis in human cancer cell lines while sparing normal cells (35, 36). However, TRAIL-based therapies tested in several clinical trials, in a broad range of different tumors, yielded very disappointing results [reviewed by Lemke et al. (37)]. Three major features are considered to limit the therapeutic activity of conventional recombinant TRAIL proteins, low *in vivo* bioactivity and short plasma half-life (38), intrinsic or acquired resistance to TRAIL (39), and inefficient delivery of the proapoptotic protein to the tumor cells, altogether requiring multiple doses with potential increase in side effects (40). In attempts to overcome these intrinsic negative features of recombinant soluble TRAIL, several studies in different tumor models, including ovary-, lung-, colon-, and pancreas-derived tumors, exploited the possibility of a transient cell-based TRAIL expression making use of MSC as delivery system because of their presumed tumor-homing potential (41–43). Despite that the specific role of MSCs in the tumor microenvironment is not fully understood, different studies reported that TRAIL expressed by MSCs can overcome resistance in colorectal and breast cancer to treatment with recombinant TRAIL (44, 45). This suggests that the advantage of using MSC-based *in situ* production of soluble TRAIL is not only favorable to overcome the short plasma half-lives of this antitumor drug but also may contribute to break TRAIL resistance of tumor cells.

Based on this knowledge, we aimed at an improvement of MSC-based drug delivery systems from two sides, the producer cell and the product itself. Thus, using a murine MSC line as a model which was shown to maintain its phenotype and differentiation potential *in vitro* and *in vivo* (28), we questioned whether instead of transient expression, stable producer clones can be obtained to lay the ground for similar approaches with human MSC and for establishment of defined drug producer cell banks suitable for allogeneic transplantation in cancer patients. Concerning the therapeutic protein, we reasoned that second-generation TRAIL molecules, with tumor targeting features and optimized apoptotic potential are functionally superior to conventional soluble TRAIL. A cell-based *in situ* expression system of the model drug, an EGFR-specific diabody single-chain TRAIL (Db-scTRAIL), comprising a hexavalent TRAIL, could be well advantageous over costly GMP expression and purification of such a complex molecule. MSCs isolated from three different sources proved to be insensitive to apoptosis induced by human recombinant TRAIL, despite the expression of DR4 and DR5 (46). Because of a low intrinsic bioactivity of such soluble TRAIL preparations, and the several orders of magnitude higher bioactivity of the Db-scTRAIL used here, we first confirmed the insensitivity of MSCs to Db-scTRAIL, even in combination with the sensitizer BZB. Thus, the model cell system used qualified for establishing a producer cell.

Concerning the transfection method, despite that the majority of the studies so far used viral vectors to genetically modify stem cells, this technology is still debated. This is due to the fact that some of these viral vectors, like lentiviruses, are immunogenic and show instability of the transgene, which can cause severe immune responses when introduced into the patients [reviewed by Stuckey and Shah (25)]. Additionally, the specific integration site of the vector DNA into the genome of the cells is crucial, and disruptions of essential genes that may cause malignant transformation have to be avoided. Despite these potential safety issues of viral transduction methods, the prospects of higher efficiency so far have favored this over non-viral transfection methods for stem cells (47, 48). Therefore, with the aim to provide alternative approaches to generate MSC drug producer cell systems, in this study, we exploited a non-viral transfection method based on PEI. We achieved the isolation of a stable and long-term expressing MSC line producing highly bioactive Db-scTRAIL under retention of its full MSC typical differentiation capability. Moreover, a significant reduction of tumor volume could be achieved already after two peritumoral administrations of MSC.TRAIL, showing that the localized production achieves therapeutically effective doses of the drug when combined with BZB, and the systemically detectable levels of the Db-scTRAIL fusion protein were well tolerated by the treated animals.

A particular feature attributed to MSCs is their potential tumor-homing capacity (9). Because we detected circulating levels of the Db-scTRAIL upon local s.c. injection of MSCs in non-tumor bearing animals, we focused in this study on analysis of macroscopic tumor regression; therefore, we cannot tell to which extent peritumorally injected MSC might have migrated to the tumor tissue and whether or not this was instrumental for the observed tumor regression. Further, the *in vivo* fate of transferred MSCs remains to be defined in future studies. Specifically parameters affecting the duration of therapeutics production, such as long-term survival with or without potential tissue-specific differentiation need to be unraveled.

Currently, the clinical application of MSC-based therapy in the regenerative medicine is widely accepted with clear benefits (49–51) while its use in oncology is still in an early exploratory phase and a general treatment concept is not yet available. Provided the model described here can be translated into a clinical application, the data obtained here suggest that with local administration of a stable producer line, aside from achieving clear tumor responses, potential additional benefits for the patients could be expected, for example concerning the frequency of therapeutic injections. In fact, in the case of TRAIL therapeutics, the standard clinical treatments but also animal tumor models with second-generation TRAIL reagents require daily or biweekly injections (19, 52). While based on the obtained results using stable producer MSCs, a regimen with a reduced frequency of administrations, weekly or even less frequent, seems achievable. Importantly, an efficient translation of cell-based therapy into clinical application often requires the ability to readily administer a safe and efficacious product at the optimal dosage. Toward this aim, an established MSC producer cell bank suited for allogeneic transplantation potentially offers enormous advantages over autologous transplantation concerning time constraints and the unclear chance of isolation of autologous MSCs from the patient suitable to use for transfection and successful protein expression. Specifically, in the context of autologous sources, patients are generally older and may present with multiple comorbidities, may impact MSC isolation and propagation both in quantitative and qualitative terms. Because of the hypo-immunogenic feature of MSCs in general, autologous transplantation appears not mandatory. Accordingly, we propose that a well-characterized stock of MSC producer lines, as the model cell line described here, is a robust alternative to cell-based expression systems relying on autologous patient material.

In conclusion, in the present study using a murine MSC line and xenografted tumors as model system, we revealed proof of concept that a stable MSC line expressing a therapeutic protein and maintaining MSC characteristics can be generated and can be applied *in vivo* to achieve a significant tumor response without apparent side effects. Our results support the exploitation of this approach for generation of stable well-characterized cell banks of human MSC producer lines for local expression of highly active, yet difficult or costly to produce, cancer therapeutics.

## Ethics Statement

Animal care and treatment were carried out in accordance with the local Ethical Committee guidelines at University of Stuttgart on the use of experimental animals and experiments approved by State authorities (Regierungspraesidium Stuttgart) under no. 35-9185.81/0413.

## Author Contributions

IM contributed to the design of the work, performed the experimental the work and data analysis, and contributed to data interpretation. MH, RK, and MS were responsible for the design and genetic engineering of Db-scTRAIL molecules and contributed to study data analyses. KP was responsible for the overall concept and design of the study, data interpretation, and the final manuscript. All the authors contributed to manuscript writing and approved the submitted manuscript.

## Conflict of Interest Statement

The authors declare that the research was conducted in the absence of any commercial or financial relationships that could be construed as a potential conflict of interest.

## Supplementary Material

Figure S1 **Scheme of the diabody single-chain TNF-related apoptosis-inducing ligand fusion protein**. An EGFR-specific scFv antibody fragment, comprising V_H_ and V_L_, was fused to the N-terminus of scTRAIL in which three extracellular TRAIL domains are genetically linked to one polypeptide chain. A peptide linker (GGGGS) between V_H_ and V_L_ was chosen to obtain a diabody configuration and, therefore, dimerization of the molecule. F, FLAG tag.

Figure S2 **Analysis of cell death induction after coculture of Colo205 with mesenchymal stem cells (MSCs)**. One day after transient transfection, MSCs (MSC.TNF-related apoptosis-inducing ligand) were cocultured with Colo205 cells (1 × 10^5^ cells) in the presence or absence of BZB (250 ng/ml) for additional 24 h. Two different ratios of MSCs and Colo205 were tested, 1:5 and 1:50. After 18 h of coculture, cell viability was analyzed by PI staining. *y*-Axis: number of events analyzed.

Figure S3 **Single clone selection**. Thirteen clones selected after Dot Blot analysis were seeded (1 × 10^6^ cells) and cultured. After 3 days, the amount of soluble diabody single-chain TNF-related apoptosis-inducing ligand released in culture media was measured by enzyme-linked immunosorbent assay. Mesenchymal stem cells untransfected (UNT) and the pool cell line were used as controls (mean ± SD, *n* = 3; ^#^, under detectable level).

Figure S4 **Diabody single-chain TNF-related apoptosis-inducing ligand (Db-scTRAIL) secreted by mesenchymal stem cell (MSC).TRAIL cell line induces apoptotic activity in HCT116**. HCT116 were **(A)** treated with serial dilutions (titration 1:3) of purified Db-scTRAIL in the absence (circles) or in the presence (squares) of 250 ng/ml of BZB or **(B)** cocultured with MSC lines (MSC:HCT116 ratios 1:5 and 1:50) in combination with BZB (250 ng/ml) and/or TRAIL blocking antibody (1 μg/ml). After 18 h, cell viability was analyzed using crystal violet staining, and data were normalized using HCT116 cells treated with BZB as control (mean ± SD, *n* = 3).

Figure S5 **Surface mesenchymal stem cells (MSC) marker expression**. Analysis of MSC marker expression at passages **(A)** 9 and **(B)** 42. Cells were stained with indicated antibodies and binding was analyzed by flow cytometry (red). Unstained cells were used as negative control (black). *y*-Axis: number of events analyzed.

Figure S6 **Analysis of diabody single-chain TNF-related apoptosis-inducing ligand (Db-scTRAIL) production *in vivo* from s.c.-injected mesenchymal stem cells (MSCs)**. MSC.TRAIL, MSCs untransfected (MSC unt), and MSC.Mock (4 × 10^6^) cells were subcutaneously injected in one flank of nude mice [*n* = 4 animals for each group, ±95% confidence interval (CI)] or 100 μl of PBS (s.c.) as control. After 1, 3, 7, 14, and 21 days serum concentration of Db-scTRAIL was analyzed by enzyme-linked immunosorbent assay assay (#, under detectable level). The groups MSC Unt, MSC.Mock, and PBS did not reveal detectable protein levels.

## References

[1] Friedenstein AJ Shapiro Piatetzky II Petrakova KV Osteogenesis in transplants of bone marrow cells. J Embryol Exp Morphol (1966) 16(3):381–90. 5336210

[2] Friedenstein AJ Petrakova KV Kurolesova AI Frolova GP Heterotopic of bone marrow. analysis of precursor cells for osteogenic and hematopoietic tissues. Transplantation (1968) 6(2):230–47. 10.1097/00007890-196803000-00009 5654088

[3] Muraglia A Cancedda R Quarto R. Clonal mesenchymal progenitors from human bone marrow differentiate in vitro according to a hierarchical model. J Cell Sci (2000) 113(Pt 7):1161–6. 10704367 10.1242/jcs.113.7.1161

[4] Aslan H Zilberman Y Kandel L Liebergall M Oskouian RJ Gazit D Osteogenic differentiation of noncultured immunoisolated bone marrow-derived Cd105+ cells. Stem Cells (2006) 24(7):1728–37. 10.1634/stemcells.2005-0546 16601078

[5] Davies LC Heldring N Kadri N Le Blanc K Mesenchymal stromal cell secretion of programmed death-1 ligands regulates T cell mediated immunosuppression. Stem Cells (2017) 35(3):766–76. 10.1002/stem.2509 27671847 PMC5599995

[6] Madec AM Mallone R Afonso G Abou Mrad E Mesnier A Eljaafari A Mesenchymal stem cells protect nod mice from diabetes by inducing regulatory T cells. Diabetologia (2009) 52(7):1391–9. 10.1007/s00125-009-1374-z 19421731

[7] Horwitz EM Prockop DJ Fitzpatrick LA Koo WW Gordon PL Neel M Transplantability and therapeutic effects of bone marrow-derived mesenchymal cells in children with osteogenesis imperfecta. Nat Med (1999) 5(3):309–13. 10.1038/6529 10086387

[8] Squillaro T Peluso G Galderisi U Clinical trials with mesenchymal stem cells: an update. Cell Transplant (2016) 25(5):829–48. 10.3727/096368915X689622 26423725

[9] Hagenhoff A Bruns CJ Zhao Y von Luttichau I Niess H Spitzweg C Harnessing mesenchymal stem cell homing as an anticancer therapy. Expert Opin Biol Ther (2016) 16(9):1079–92. 10.1080/14712598.2016.1196179 27270211

[10] Niess H von Einem JC Thomas MN Michl M Angele MK Huss R Treatment of advanced gastrointestinal tumors with genetically modified autologous mesenchymal stromal cells (treat-Me1): study protocol of a phase I/II clinical trial. BMC Cancer (2015) 15:237. 10.1186/s12885-015-1241-x 25879229 PMC4393860

[11] Nowakowski A Drela K Rozycka J Janowski M Lukomska B. Engineered mesenchymal stem cells as an anti-cancer trojan horse. Stem Cells Dev (2016) 25(20):1513–31. 10.1089/scd.2016.0120 27460260 PMC5035934

[12] Carswell EA Old LJ Kassel RL Green S Fiore N Williamson B. An endotoxin-induced serum factor that causes necrosis of tumors. Proc Natl Acad Sci U S A (1975) 72(9):3666–70. 10.1073/pnas.72.9.3666 1103152 PMC433057

[13] Herbst RS Eckhardt SG Kurzrock R Ebbinghaus S O’Dwyer PJ Gordon MS Phase I dose-escalation study of recombinant human Apo2l/trail, a dual proapoptotic receptor agonist, in patients with advanced cancer. J Clin Oncol (2010) 28(17):2839–46. 10.1200/JCO.2009.25.1991 20458040

[14] Soria JC Smit E Khayat D Besse B Yang X Hsu CP Phase 1b study of dulanermin (recombinant human Apo2l/trail) in combination with paclitaxel, carboplatin, and bevacizumab in patients with advanced non-squamous non-small-cell lung cancer. J Clin Oncol (2010) 28(9):1527–33. 10.1200/JCO.2009.25.4847 20159815

[15] Roberts NJ Zhou S Diaz LA Jr Holdhoff M Systemic use of tumor necrosis factor alpha as an anticancer agent. Oncotarget (2011) 2(10):739–51. 10.18632/oncotarget.344 22036896 PMC3248159

[16] Ganten TM Koschny R Sykora J Schulze-Bergkamen H Buchler P Haas TL Preclinical differentiation between apparently safe and potentially hepatotoxic applications of trail either alone or in combination with chemotherapeutic drugs. Clin Cancer Res (2006) 12(8):2640–6. 10.1158/1078-0432.CCR-05-2635 16638878

[17] Muller N Schneider B Pfizenmaier K Wajant H. Superior serum half life of albumin tagged Tnf ligands. Biochem Biophys Res Commun (2010) 396(4):793–9. 10.1016/j.bbrc.2010.04.134 20447380

[18] Schneider B Munkel S Krippner-Heidenreich A Grunwald I Wels WS Wajant H Potent antitumoral activity of trail through generation of tumor-targeted single-chain fusion proteins. Cell Death Dis (2010) 1:e68. 10.1038/cddis.2010.45 21364672 PMC3032523

[19] Siegemund M Seifert O Zarani M Dzinic T De Leo V Gottsch D An optimized antibody-single-chain trail fusion protein for cancer therapy. MAbs (2016) 8(5):879–91. 10.1080/19420862.2016.1172163 27064440 PMC4968136

[20] Guiho R Biteau K Grisendi G Taurelle J Chatelais M Gantier M Trail delivered by mesenchymal stromal/stem cells counteracts tumor development in orthotopic ewing sarcoma models. Int J Cancer (2016) 139(12):2802–11. 10.1002/ijc.30402 27558972

[21] Yan C Song X Yu W Wei F Li H Lv M Human umbilical cord mesenchymal stem cells delivering strail home to lung cancer mediated by Mcp-1/Ccr2 axis and exhibit antitumor effects. Tumour Biol (2016) 37(6):8425–35. 10.1007/s13277-015-4746-7 26733169

[22] Lathrop MJ Sage EK Macura SL Brooks EM Cruz F Bonenfant NR Antitumor effects of trail-expressing mesenchymal stromal cells in a mouse xenograft model of human mesothelioma. Cancer Gene Ther (2015) 22(1):44–54. 10.1038/cgt.2014.68 25525034

[23] Compte M Cuesta AM Sanchez-Martin D Alonso-Camino V Vicario JL Sanz L Tumor immunotherapy using gene-modified human mesenchymal stem cells loaded into synthetic extracellular matrix scaffolds. Stem Cells (2009) 27(3):753–60. 10.1634/stemcells.2008-0831 19096041 PMC2729675

[24] Zhang X Yang Y Zhang L Lu Y Zhang Q Fan D Mesenchymal stromal cells as vehicles of tetravalent bispecific tandab (Cd3/Cd19) for the treatment of B cell lymphoma combined with Ido pathway inhibitor d-1-methyl-tryptophan. J Hematol Oncol (2017) 10(1):56. 10.1186/s13045-017-0397-z 28228105 PMC5322661

[25] Stuckey DW Shah K. Trail on trial: preclinical advances in cancer therapy. Trends Mol Med (2013) 19(11):685–94. 10.1016/j.molmed.2013.08.007 24076237 PMC3880796

[26] Karantalis V Hare JM Use of mesenchymal stem cells for therapy of cardiac disease. Circ Res (2015) 116(8):1413–30. 10.1161/CIRCRESAHA.116.303614 25858066 PMC4429294

[27] Hare JM DiFede DL Rieger AC Florea V Landin AM El-Khorazaty J Randomized comparison of allogeneic versus autologous mesenchymal stem cells for nonischemic dilated cardiomyopathy: poseidon-Dcm trial. J Am Coll Cardiol (2017) 69(5):526–37. 10.1016/j.jacc.2016.11.009 27856208 PMC5291766

[28] Raeth S Sacchetti B Siegel G Mau-Holzmann UA Hansmann J Vacun G A mouse bone marrow stromal cell line with skeletal stem cell characteristics to study osteogenesis in vitro and in vivo. Stem Cells Dev (2014) 23(10):1097–108. 10.1089/scd.2013.0367 24405418

[29] Rapp AE Bindl R Heilmann A Erbacher A Müller I Brenner RE Systemic mesenchymal stem cell administration enhances bone formation in fracture repair but not load-induced bone formation. Eur Cell Mater (2015) 29:22–34. 25552426 10.22203/ecm.v029a02

[30] Siegemund M Pollak N Seifert O Wahl K Hanak K Vogel A Superior antitumoral activity of dimerized targeted single-chain trail fusion proteins under retention of tumor selectivity. Cell Death Dis (2012) 3:e295. 10.1038/cddis.2012.29 22495350 PMC3358007

[31] Dominici M Le Blanc K Mueller I Slaper-Cortenbach I Marini F Krause D Minimal criteria for defining multipotent mesenchymal stromal cells. The international society for cellular therapy position statement. Cytotherapy (2006) 8(4):315–7. 10.1080/14653240600855905 16923606

[32] Boxall SA Jones E. Markers for characterization of bone marrow multipotential stromal cells. Stem Cells Int (2012) 2012:975871. 10.1155/2012/975871 22666272 PMC3361338

[33] Wiley SR Schooley K Smolak PJ Din WS Huang CP Nicholl JK Identification and characterization of a new member of the Tnf family that induces apoptosis. Immunity (1995) 3(6):673–82. 10.1016/1074-7613(95)90057-8 8777713

[34] Pitti RM Marsters SA Ruppert S Donahue CJ Moore A Ashkenazi A. Induction of apoptosis by Apo-2 ligand, a new member of the tumor necrosis factor cytokine family. J Biol Chem (1996) 271(22):12687–90. 10.1074/jbc.271.22.12687 8663110

[35] Ashkenazi A Pai RC Fong S Leung S Lawrence DA Marsters SA Safety and antitumor activity of recombinant soluble Apo2 ligand. J Clin Invest (1999) 104(2):155–62. 10.1172/JCI6926 10411544 PMC408479

[36] Walczak H Miller RE Ariail K Gliniak B Griffith TS Kubin M Tumoricidal activity of tumor necrosis factor-related apoptosis-inducing ligand in vivo. Nat Med (1999) 5(2):157–63. 10.1038/5517 9930862

[37] Lemke J von Karstedt S Zinngrebe J Walczak H. Getting trail back on track for cancer therapy. Cell Death Differ (2014) 21(9):1350–64. 10.1038/cdd.2014.81 24948009 PMC4131183

[38] Micheau O Shirley S Dufour F. Death receptors as targets in cancer. Br J Pharmacol (2013) 169(8):1723–44. 10.1111/bph.12238 23638798 PMC3753832

[39] Maksimovic-Ivanic D Stosic-Grujicic S Nicoletti F Mijatovic S. Resistance to trail and how to surmount it. Immunol Res (2012) 52(1–2):157–68. 10.1007/s12026-012-8284-8 22407575

[40] Kelley SK Harris LA Xie D Deforge L Totpal K Bussiere J Preclinical studies to predict the disposition of Apo2l/tumor necrosis factor-related apoptosis-inducing ligand in humans: characterization of in vivo efficacy, pharmacokinetics, and safety. J Pharmacol Exp Ther (2001) 299(1):31–8. 11561060

[41] Yu R Deedigan L Albarenque SM Mohr A Zwacka RM. Delivery of strail variants by Mscs in combination with cytotoxic drug treatment leads to P53-independent enhanced antitumor effects. Cell Death Dis (2013) 4:e503. 10.1038/cddis.2013.19 23429289 PMC3734822

[42] Yuan Z Kolluri KK Sage EK Gowers KH Janes SM. Mesenchymal stromal cell delivery of full-length tumor necrosis factor-related apoptosis-inducing ligand is superior to soluble type for cancer therapy. Cytotherapy (2015) 17(7):885–96. 10.1016/j.jcyt.2015.03.603 25888191 PMC4503823

[43] Grisendi G Spano C D’Souza N Rasini V Veronesi E Prapa M Mesenchymal progenitors expressing trail induce apoptosis in sarcomas. Stem Cells (2015) 33(3):859–69. 10.1002/stem.1903 25420617

[44] Mueller LP Luetzkendorf J Widder M Nerger K Caysa H Mueller T. Trail-transduced multipotent mesenchymal stromal cells (trail-Msc) overcome trail resistance in selected Crc cell lines in vitro and in vivo. Cancer Gene Ther (2011) 18(4):229–39. 10.1038/cgt.2010.68 21037557

[45] Lin T Huang X Gu J Zhang L Roth JA Xiong M Long-term tumor-free survival from treatment with the Gfp-trail fusion gene expressed from the Htert promoter in breast cancer cells. Oncogene (2002) 21(52):8020–8. 10.1038/sj.onc.1205926 12439752

[46] Szegezdi E O’Reilly A Davy Y Vawda R Taylor DL Murphy M Stem cells are resistant to trail receptor-mediated apoptosis. J Cell Mol Med (2009) 13(11–12):4409–14. 10.1111/j.1582-4934.2008.00522.x 19604313 PMC4515056

[47] Choi SA Hwang SK Wang KC Cho BK Phi JH Lee JY Therapeutic efficacy and safety of trail-producing human adipose tissue-derived mesenchymal stem cells against experimental brainstem glioma. Neuro Oncol (2011) 13(1):61–9. 10.1093/neuonc/noq147 21062796 PMC3018907

[48] Hu YL Huang B Zhang TY Miao PH Tang GP Tabata Y Mesenchymal stem cells as a novel carrier for targeted delivery of gene in cancer therapy based on nonviral transfection. Mol Pharm (2012) 9(9):2698–709. 10.1021/mp300254s 22862421

[49] Stanovici J, Le Nail LR Brennan MA Vidal L Trichet V Rosset P Bone regeneration strategies with bone marrow stromal cells in orthopaedic surgery. Curr Res Transl Med (2016) 64(2):83–90. 10.1016/j.retram.2016.04.006 27316391

[50] Fellows CR Matta C Zakany R Khan IM Mobasheri A. Adipose, bone marrow and synovial joint-derived mesenchymal stem cells for cartilage repair. Front Genet (2016) 7:213. 10.3389/fgene.2016.00213 28066501 PMC5167763

[51] Prè ED Conti G Sbarbati A. Hyaluronic acid (Ha) scaffolds and multipotent stromal cells (Mscs) in regenerative medicine. Stem Cell Rev (2016) 12(6):664–81. 10.1007/s12015-016-9684-2 27665291

[52] Fleten KG Florenes VA Prasmickaite L Hill O Sykora J Maelandsmo GM Hvtra, a novel trail receptor agonist, induces apoptosis and sustained growth retardation in melanoma. Cell Death Discov (2016) 2:16081 10.1038/cddiscovery.2016.81 28028438 PMC5149582

