# Supplementary figures and images for: Antitumor Activity of a Mesenchymal Stem Cell Line Stably Secreting a Tumor-Targeted TNF-Related Apoptosis-Inducing Ligand Fusion Protein
Source: Front Immunol. 2017 May 11;8:536. doi: 10.3389/fimmu.2017.00536 (PMC5425590; doi:10.3389/fimmu.2017.00536)

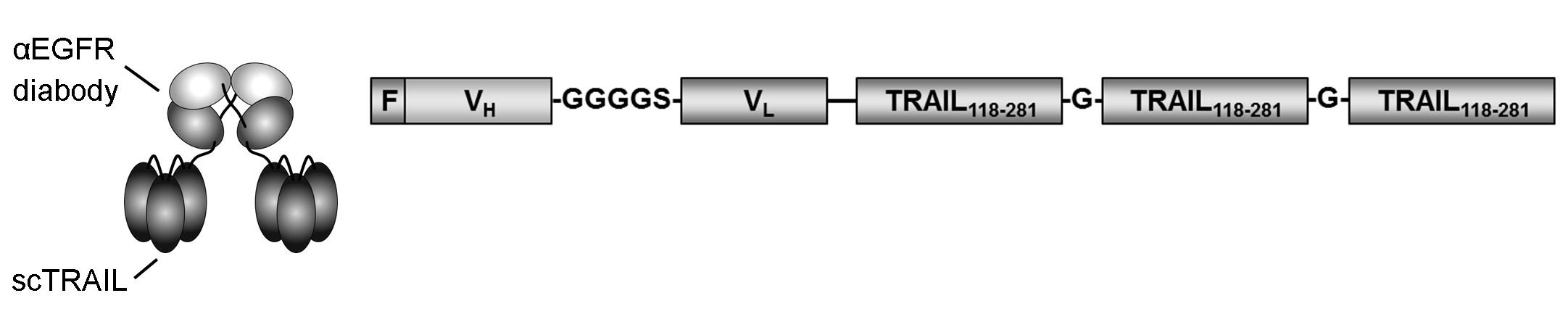

Supplement: Figure S1 — Scheme of the diabody single-chain TNF-related apoptosis-inducing ligand fusion protein. An EGFR-specific scFv antibody fragment, comprising VH and VL, was fused to the N-terminus of scTRAIL in which three extracellular TRAIL domains are genetically linked to one polypeptide chain. A peptide linker (GGGGS) between VH and VL was chosen to obtain a diabody configuration and, therefore, dimerization of the molecule. F, FLAG tag. [file Image_1.TIF]

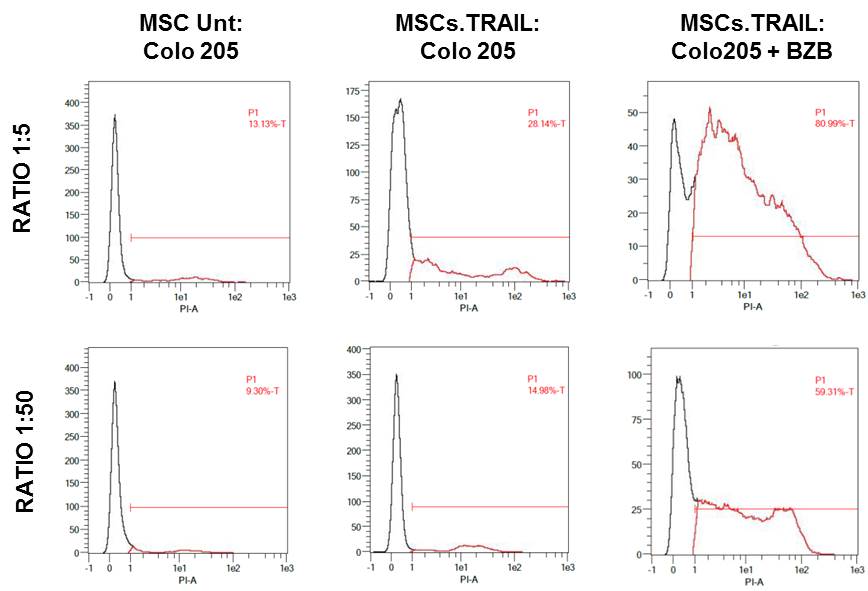

Supplement: Figure S2 — Analysis of cell death induction after coculture of Colo205 with mesenchymal stem cells (MSCs). One day after transient transfection, MSCs (MSC.TNF-related apoptosis-inducing ligand) were cocultured with Colo205 cells (1 × 10^5^ cells) in the presence or absence of BZB (250 ng/ml) for additional 24 h. Two different ratios of MSCs and Colo205 were tested, 1:5 and 1:50. After 18 h of coculture, cell viability was analyzed by PI staining. y-Axis: number of events analyzed. [file Image_2.JPEG]

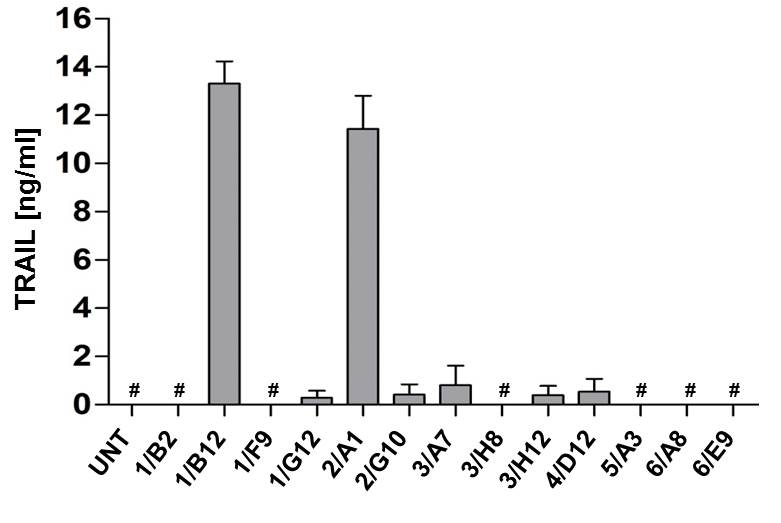

Supplement: Figure S3 — Single clone selection. Thirteen clones selected after Dot Blot analysis were seeded (1 × 10^6^ cells) and cultured. After 3 days, the amount of soluble diabody single-chain TNF-related apoptosis-inducing ligand released in culture media was measured by enzyme-linked immunosorbent assay. Mesenchymal stem cells untransfected (UNT) and the pool cell line were used as controls (mean ± SD, n = 3; #, under detectable level). [file Image_3.JPEG]

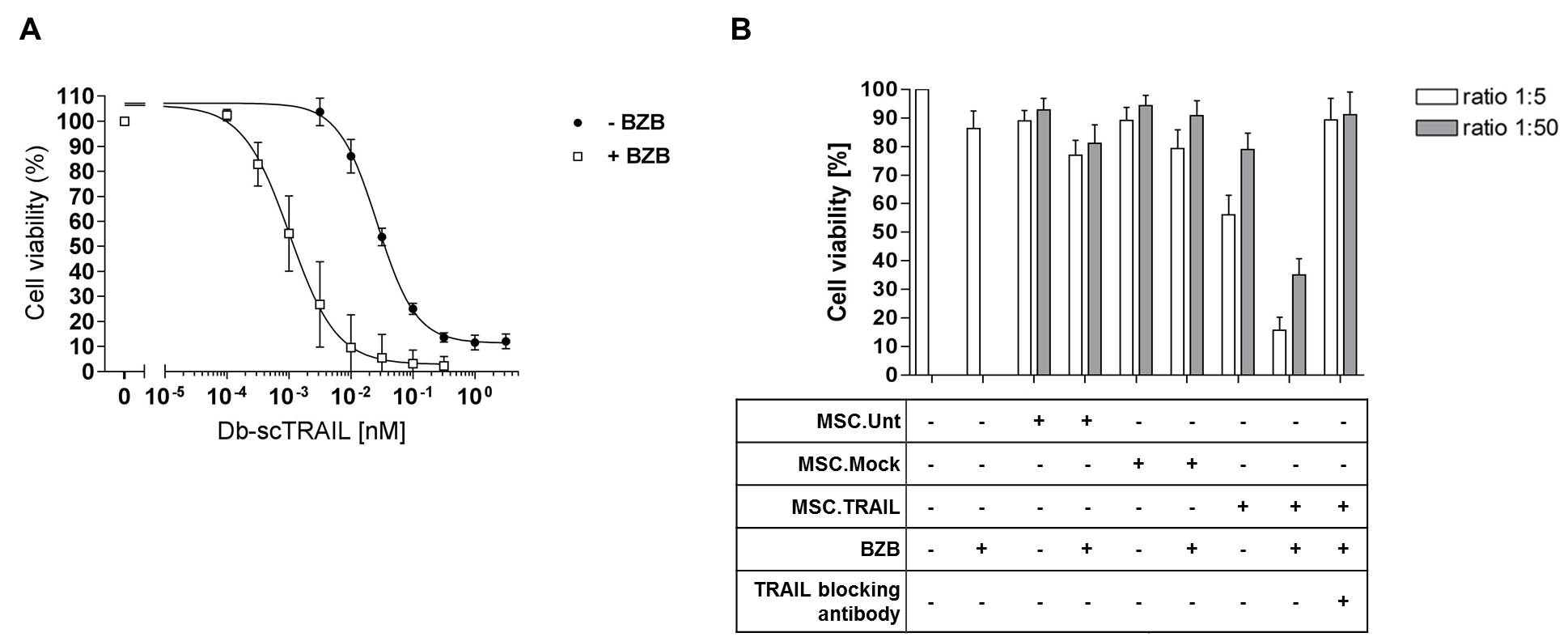

Supplement: Figure S4 — Diabody single-chain TNF-related apoptosis-inducing ligand (Db-scTRAIL) secreted by mesenchymal stem cell (MSC).TRAIL cell line induces apoptotic activity in HCT116. HCT116 were (A) treated with serial dilutions (titration 1:3) of purified Db-scTRAIL in the absence (circles) or in the presence (squares) of 250 ng/ml of BZB or (B) cocultured with MSC lines (MSC:HCT116 ratios 1:5 and 1:50) in combination with BZB (250 ng/ml) and/or TRAIL blocking antibody (1 μg/ml). After 18 h, cell viability was analyzed using crystal violet staining, and data were normalized using HCT116 cells treated with BZB as control (mean ± SD, n = 3). [file Image_4.JPEG]

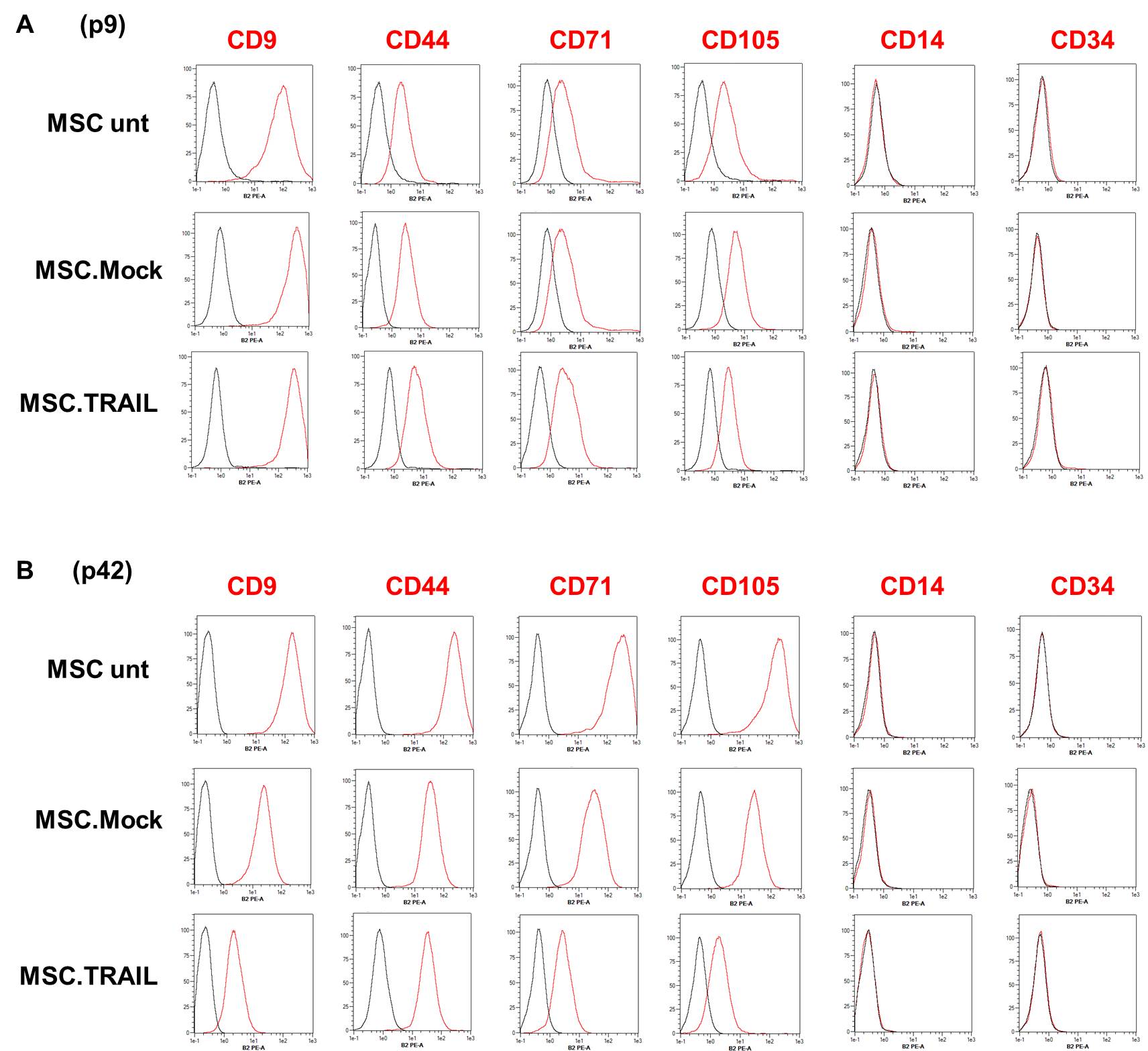

Supplement: Figure S5 — Surface mesenchymal stem cells (MSC) marker expression. Analysis of MSC marker expression at passages (A) 9 and (B) 42. Cells were stained with indicated antibodies and binding was analyzed by flow cytometry (red). Unstained cells were used as negative control (black). y-Axis: number of events analyzed. [file Image_5.JPEG]

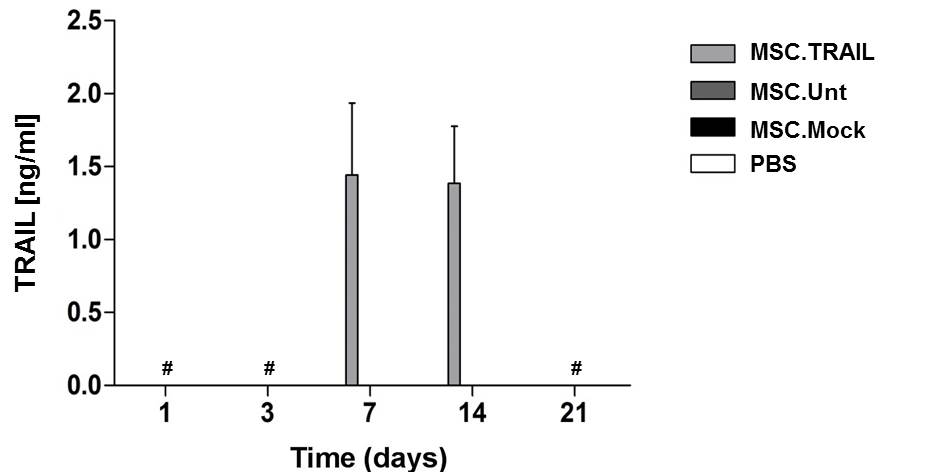

Supplement: Figure S6 — Analysis of diabody single-chain TNF-related apoptosis-inducing ligand (Db-scTRAIL) production in vivo from s.c.-injected mesenchymal stem cells (MSCs). MSC.TRAIL, MSCs untransfected (MSC unt), and MSC.Mock (4 × 10^6^) cells were subcutaneously injected in one flank of nude mice [n = 4 animals for each group, ±95% confidence interval (CI)] or 100 μl of PBS (s.c.) as control. After 1, 3, 7, 14, and 21 days serum concentration of Db-scTRAIL was analyzed by enzyme-linked immunosorbent assay assay (#, under detectable level). The groups MSC Unt, MSC.Mock, and PBS did not reveal detectable protein levels. [file Image_6.JPEG]
